# Whooping crane use of riverine stopover sites

**DOI:** 10.1371/journal.pone.0209612

**Published:** 2019-01-09

**Authors:** David M. Baasch, Patrick D. Farrell, Shay Howlin, Aaron T. Pearse, Jason M. Farnsworth, Chadwin B. Smith

**Affiliations:** 1 Executive Director’s Office for the Platte River Recovery Implementation Program, Kearney, NE, United States of America; 2 Western Ecosystems Technology, Inc. (WEST), Cheyenne, Wyoming, United States of America; 3 U.S. Geological Survey, Northern Prairie Wildlife Research Center, Jamestown, North Dakota, United States of America; University of Tulsa, UNITED STATES

## Abstract

Migratory birds like endangered whooping cranes (*Grus americana*) require suitable nocturnal roost sites during twice annual migrations. Whooping cranes primarily roost in shallow surface water wetlands, ponds, and rivers. All these features have been greatly impacted by human activities, which present threats to the continued recovery of the species. A portion of one such river, the central Platte River, has been identified as critical habitat for the survival of the endangered whooping crane. Management intervention is now underway to rehabilitate habitat form and function on the central Platte River to increase use and thereby contribute to the survival of whooping cranes. The goal of our analyses was to develop habitat selection models that could be used to direct riverine habitat management activities (i.e., channel widening, tree removal, flow augmentation, etc.) along the central Platte River and throughout the species’ range. As such, we focused our analyses on two robust sets of whooping crane observations and habitat metrics the Platte River Recovery Implementation Program (Program or PRRIP) and other such organizations could influence. This included channel characteristics such as total channel width, the width of channel unobstructed by dense vegetation, and distance of forest from the edge of the channel and flow-related metrics like wetted width and unit discharge (flow volume per linear meter of wetted channel width) that could be influenced by flow augmentation or reductions during migration. We used 17 years of systematic monitoring data in a discrete-choice framework to evaluate the influence these various metrics have on the relative probability of whooping crane use and found the width of channel unobstructed by dense vegetation and distance to the nearest forest were the best predictors of whooping crane use. Secondly, we used telemetry data obtained from a sample of 38 birds of all ages over the course of seven years, 2010–2016, to evaluate whooping crane use of riverine habitat within the North-central Great Plains, USA. For this second analysis, we focused on the two metrics found to be important predictors of whooping crane use along the central Platte River, unobstructed channel width and distance to nearest forest or wooded area. Our findings indicate resource managers, such as the Program, have the potential to influence whooping crane use of the central Platte River through removal of in-channel vegetation to increase the unobstructed width of narrow channels and through removal of trees along the bank line to increase unforested corridor widths. Results of both analyses also indicated that increases in relative probability of use by whooping cranes did not appreciably increase with unobstructed views ≥200 m wide and unforested corridor widths that were ≥330 m. Therefore, managing riverine sites for channels widths >200 m and removing trees beyond 165 m from the channel’s edge would increase costs associated with implementing management actions such as channel and bank-line disking, removing trees, augmenting flow, etc. without necessarily realizing an additional appreciable increase in use by migrating whooping cranes.

## Introduction

Each year, whooping cranes of the Aransas–Wood Buffalo (AWB) population undertake two 3,900-kilometer migrations between breeding areas in and around Wood Buffalo National Park in Canada and wintering areas in and around Aransas National Wildlife Area on the Gulf Coast of Texas, USA. The migration route is well documented and the vast majority of whooping crane observations occur within an approximately 300-kilometer wide corridor through Alberta, Saskatchewan, Montana, North Dakota, South Dakota, Nebraska, Kansas, Oklahoma, and Texas [1]. During migration, whooping cranes, like most migratory birds, require stopover sites to rest and build energy reserves to successfully complete migration [2]. Although a variety of habitats are used during migration, surface water is generally associated with stopover sites, where whooping cranes typically roost standing in shallow water associated with palustrine or lacustrine wetlands and river channels [3]. However, impacts of water and land development in the migration path has led to concern about the quality and quantity of stopover habitat for roosting and foraging [4]. For example, wetland loss in U.S. states in the Great Plains has been well documented with estimated reduction of wetlands ranging from 35 to 67% over the past century [5]. In addition, water development structures such as dams have been and continue to be installed to retime water releases for irrigation, power generation, and other uses which has the potential to impact riverine habitats that migratory species like whooping cranes depend on [4].

Whooping crane stopovers last from one to several days during migrations that can last several weeks [1, 3, 6]. At stopover sites, whooping cranes generally roost standing in shallow water associated with palustrine, lacustrine, or riverine wetlands. Riverine sites have been estimated to represent between 19 and 22% of roosting sites used by whooping cranes [2], but river sites have received considerable conservation attention because one of three critical habitat designations under the Endangered Species Act in the migration corridor was designated at the Big Bend reach of the central Platte River in Nebraska [7–9]. The National Research Council [4] supported this critical habitat designation and concluded that habitat conditions along the central Platte River at that time adversely affect the likelihood of survival and recovery of the whooping crane population, although the Platte River is only one of many stopover sites that whooping crane use and require during migration [1]. Consequently, characteristics of central Platte River roost habitat have been examined and described in detail [10–14]. Most analyses conducted to date have focused on evaluations of metrics such as channel width, flow, etc. presumed to be important for whooping crane habitat selection [8,9,11]. These analyses, however, have generally been developed based on a limited amount of quantitative information and most criteria for suitable roosting habitat have been derived from circumstantial roost locations based on the U.S. Fish and Wildlife Service’s opportunistic sightings database [7,11,15–17].

Early examinations of roost sites on the central Platte River identified wide, unvegetated channels and open visibility with the absence of tall trees or dense shrubs near the roost as important habitat characteristics [4,10,12,18–22]. More recent evaluations of riverine roost site habitat characteristics along the central Platte River have largely been focused on geomorphic and hydrologic metrics including unobstructed channel width, distance to obstruction (e.g., nearest forest), wetted width, area of suitable depth, and flow [8,13]. These characterizations, however, generally were not based on robust analyses of empirical data and thus often reflect the investigators assumptions about the habitat metrics that drive whooping crane roost site selection and are potentially influenced by sampling bias, detection bias, and location error due to the opportunistic nature of the data collection process [7]. Sampling bias can result when opportunistic sampling methods are employed and could affect results of analyses if whooping crane groups were not detected, reported, or confirmed with equal probabilities across the landscape [7]. For example, if whooping cranes are more likely to be detected at a wildlife sanctuary that has thousands of viewers and is managed for wide open channels, but they occur equally as often in areas off of the sanctuary with narrow channels where they are not detected, results of analyses would indicate they select wide channels which would be a biased representation of reality.

We used whooping crane stopover data collected via systematic aerial surveys over the course of 17 years and a discrete-choice framework to evaluate whooping crane use of riverine habitat along the central Platte River. This was accomplished through an evaluation of channel and flow habitat characteristics at systematically detected whooping crane group stopover locations within a use-available resource selection estimation framework. Next, we used telemetry data obtained from a sample of whooping cranes of all ages over 13 migration seasons, 2010–2016, to evaluate whooping crane use of riverine habitat throughout the North-central Great Plains. The objective of our analyses was to investigate riverine habitat selection by whooping cranes using methods that allow us to identify habitat metrics that are both important for whooping crane use and that can be influenced through management activities. We aimed to achieve this in a manner that addressed changes in habitat throughout our study periods and potential biases associated with evaluations of circumstantial or opportunistic roost locations.

## Methods

**Fig 1:**
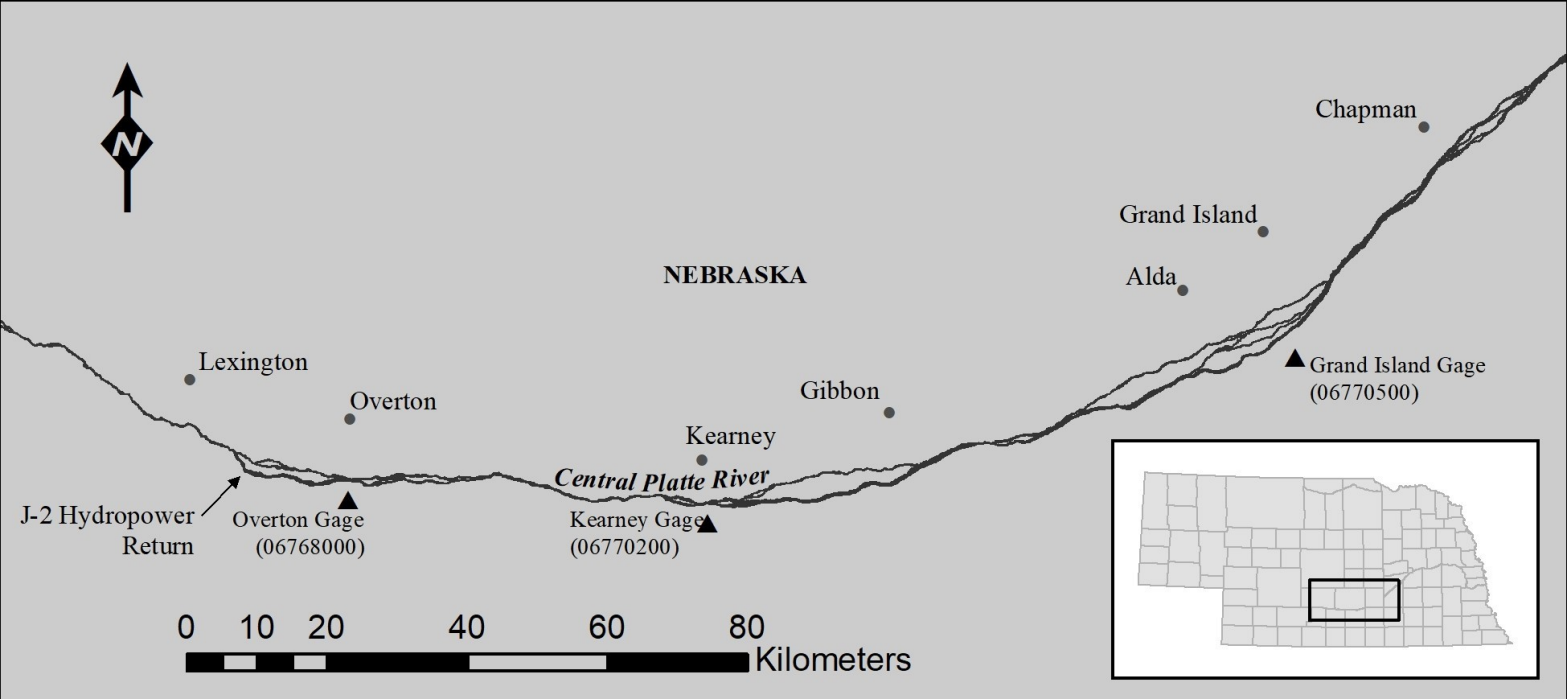
Associated Habitat Reach of the central Platte River extending from Lexington downstream to Chapman, NE.

Our first study area, the Program Associated Habitat Reach (AHR), encompassed Platte River channels and a 5.6-km buffer adjacent to the channel from the junction of US Highway 283 and Interstate 80 (near Lexington, Nebraska) downstream to Chapman, Nebraska (Fig 1). Systematic whooping crane use data was collected during the spring and fall migration periods per the Program’s whooping crane monitoring protocol [23]. Aerial surveys were flown daily from east to west at a targeted elevation of 330 m and speed of 150 km per hour. Two flights were flown each day with the east flight covering Chapman, Nebraska to the Highway 10-Platte River bridge and the west flight covering between the Highway 10-Platte River bridge and Lexington, Nebraska. The spring monitoring period spanned from March 21 to April 29 and the fall monitoring period spanned from October 9 to November 10 each year. Flights followed the main river channel and took place at dawn to locate crane groups before they departed the river to begin foraging at off-channel sites. Return flights occurred after the river survey was completed to systematically survey upland areas and smaller side channels. When a whooping crane group was detected, photographs that included the surrounding landscape were taken so the precise location of the group could be determined by pinpointing surrounding bank line and island features in a geographic information system and aerial imagery that was collected annually. In addition, decoys were placed at random locations within the channel each migration season to determine whether or not channel or flow characteristics influenced detection of whooping crane groups.

**Fig 2:**
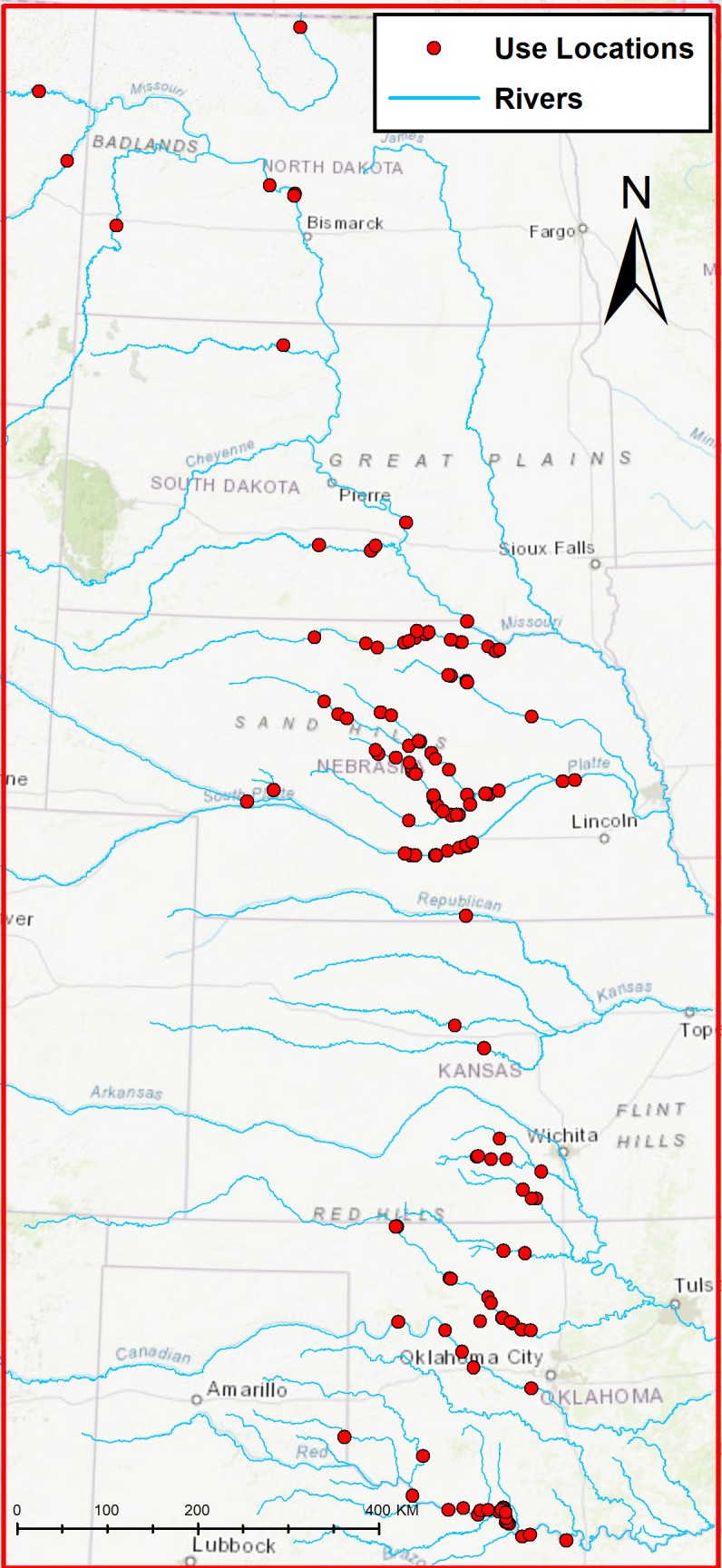
Great Plains study area including riverine use locations (points) included in our analysis.

Our second study area included the migration corridor for the Aransas–Wood Buffalo population within Montana, North Dakota, South Dakota, Nebraska, Kansas, Oklahoma, and Texas (Fig 2). Locational data (henceforth, telemetry data) were gathered from 68 GPS-marked whooping cranes, spring 2010 –spring 2016 [1]. In test of locational accuracy, we found the median distance between a known location and the location retrieved from transmitters was 9 m [3]. For this work, we used a subset of data including stopover (use) locations that occurred in riverine habitat (wetted channels) within the study area. To describe used sites, we included a single location recorded during the first night of the stopover for each whooping crane and stopover site (i.e., multi-day stopovers were included once in the analysis). When >1 radio-marked whooping crane was present at a stopover at the same time, we only included a single use location for one randomly selected bird present at the stopover site. We defined stopover sites as locations occupied by cranes as evening roosts for ≥1 night.

### Parameterization of the *a priori* model set

Hydrologic metrics, such as wetted width and area of suitable depth, are highly dependent on instantaneous flow and change continuously while, without intervention, geomorphic metrics generally change over longer periods of time (i.e., years). Given the relative stability of geomorphic features, we were able to obtain accurate estimates of unobstructed channel width (UOCW), total channel width (TCW), and nearest forest (NF) remotely. However, the variability in hydrologic metrics such as area of suitable depth and wetted width required hydraulic modeling to calculate more stable and estimable metrics including unit discharge (UD) and discharge divided by total channel width (DIS). Unit discharge was calculated as total discharge divided by the wetted width of the active channel. Whooping crane selection for increasing UD would generally equate to an increase in wetted width and depth. We evaluated discharge divided by total channel width, which relates flow to the total width of all channels. This metric was evaluated because total channel width can more readily be managed than the wetted width of the channel at a specific discharge.

**Fig 3:**
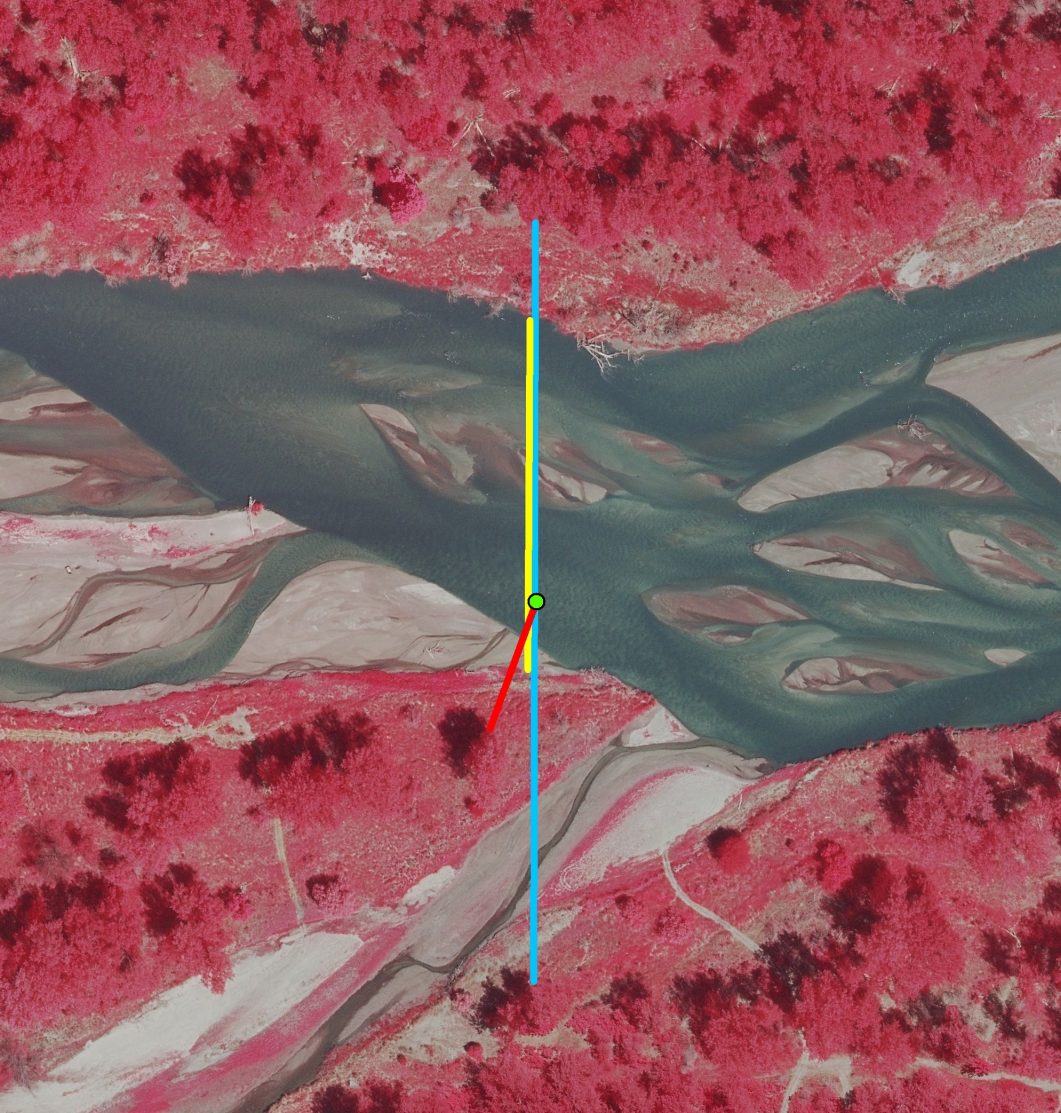
Example of how unobstructed channel width (UOCW; yellow lines), nearest forest (NF; red lines) and unforested channel width (UFCW; blue lines) were measured at whooping crane use and available locations.

We quantified the characteristics of in-channel riverine habitat with two basic sources of information: aerial imagery and a HEC-RAS hydraulic model. We used aerial photographs and remote sensing data from LiDAR to determine the following metrics of channel openness for the analysis (Fig 3):

- Unobstructed channel width (UOCW)—Width of channel unobstructed by dense vegetation.
- Nearest forest (NF)—Distance to nearest riparian forest, capped at 400 m.
- Unforested channel width (UFCW)—Width of channel unobstructed by riparian forest.

We ran the Program’s system scale HEC-RAS hydraulic model using the mean daily discharge at the nearest stream gage on the date of each whooping crane group observation to calculate the following metrics to describe flow-related channel characteristics:

- Total channel width (TCW)—Total width of channel from left bank to right bank
- Unit discharge (UD)—Flow (cms) per linear meter of wetted channel width.
- Discharge divided by total channel width (DIS)—Flow (cms) per linear meter of total channel width.

The input HEC-RAS model geometry was developed primarily using 2009 LiDAR topography supplemented with 2009 surveyed channel transects and longitudinal profile surveys. Model roughness values were based on 2005 land use dataset. The model was calibrated based on gage rating curves, March 2009 inferred water surface elevation from LiDAR data, and 2009 surveyed water surface elevation.

We used results of the analyses of central Platte River data to parameterize an *a priori* set of models that were used to evaluate whooping crane habitat selection throughout the North-central Great Plains. Specifically, we evaluated the influence of distance to nearest forest (NF) and unobstructed channel width (UOCW) on habitat selection by whooping cranes throughout the North-central Great Plains.

A GIS and USDA-NRCS Geospatial Imagery Data (Available at: https://datagateway.nrcs.usda.gov/) were used to delineate unobstructed width of channels along a line running perpendicular to the channel and through each stopover and available location. Unobstructed channel width (UOCW) was defined as the width of channel lacking dense vegetation as observed in USDA-NRCS Geospatial Imagery Data collected closest to the migration season in which the use occurred. Remote measurements using geospatial imagery data has been found to closely approximate on-the-ground physical measurements of unobstructed channel width measurements [24]. When channels were segmented by a densely-vegetated island, UOCW was delineated based on the portion of channel the stopover or available location was contained within. Distance to nearest forest or wooded area (NF) was defined as the distance from the use or available location to the nearest forested area. Distance to nearest forest was truncated at 400 m when no wooded area was located within 400 m of the use or available location because distances beyond this were deemed to have no influence on whooping crane use.

### Defining the available choice set

As an aerially migrating whooping crane group approaches the river it was assumed it cannot visually see the entire 145-km length of the AHR. Consequently, the choice set for each stopover location were necessarily limited to a subsection of the AHR. The choice set represents a sample of points from an area the crane group could have selected for use. In the discrete-choice framework, the choice set is unique for each choice, or use location. In effect, the model allows the comparison between characteristics of each use location and the characteristics of the choice set. For the purposes of this analysis, we limited the choice set to a 32-km reach of river centered on the use location and extending 16 km upstream and downstream from that point. This decision was based on an aerial evaluation of viewsheds from 915 m above ground level, the reported elevation for long distance flights by telemetry-marked whooping cranes in the 1980s [25] which has also been a commonly observed migration elevation during the recent telemetry study (PRRIP unpublished data). At 915 m above ground, only large features such as bridge crossings were readily discernable at distances >16 km from the flight location without supplemental magnification. McFadden [26] and Baasch et al. [27] found including 5 random locations per use location resulted in stable and reliable estimates of resource use; however, Baasch et al. [27] also found increasing the number of random locations resulted in improved model fit when the area defined to be available was misclassified. For these reasons, we used Hawth’s Tools [28] to randomly generate 20 available locations per stopover location within each 32-km river segment. When paired with the use location, each set of points represented a single choice set.

Habitat metrics were calculated for each whooping crane group use location and at the 20 corresponding randomly selected in-channel available points within 16 km upstream and downstream of the use location. Sixteen *a priori* candidate models, including a null model, were developed based on the habitat variables described above (Table 1). Habitat metrics were not included in the same model if their Pearson’s product moment correlation coefficient was high (|r| >0.6). This set of models, with inclusion of a null model containing no habitat metrics, composed the complete set of *a priori* models evaluated (Table 1). Akaike Information Criterion (AIC) statistic was used in the model selection process to determine which *a priori* model was most parsimonious and useful in predicting habitat use [29]. The most parsimonious *a priori* model with a ΔAIC ≤2.0 was used for inference regarding habitat selection [29].

**Table 1:** In-channel riverine *a priori* model list evaluated for whooping crane roosting habitat use along the central Platte River. The interpretation assumes an *a priori* direction (positive or negative) in the relationship between whooping crane habitat use and metrics, but actual model fit based on data could have been in the opposite direction.

Model	*A priori* Models	Interpretation
1	NULL	Habitat selection is random
2	UOCW	Select channels with views unobstructed by dense vegetation or wooded islands.
3	TCW	Select channels with increased distance from right to left bank including vegetated and wooded islands.
4	NF	Select channels with increased ‘openness’ which includes areas without trees located nearby in any direction.
5	UF	Select channels with wide unforested widths.
6	UD	Selection for amount of flow (cms) per unit of wetted channel width (m) provides suitable conditions for use.
7	DIS	Selection for amount of flow (cms) per unit of total channel width (m) provides suitable conditions for use.
8	UOCW+NF	Select channels with views unobstructed by dense vegetation or wooded islands and with increased ‘openness’ which includes areas without trees located nearby in any direction.
9	UOCW+UD	Select channels with views unobstructed by dense vegetation and amount of flow (cms) per unit of total channel width (m) provides suitable conditions for use.
10	UOCW+DIS	Select channels with views unobstructed by dense vegetation and amount of flow (cms) per unit of total channel width (m) provides suitable conditions for use.
11	TCW+UOCW	Select channels with views unobstructed by dense vegetation or wooded islands and increased distance from right to left bank that can include vegetated and wooded islands.
12	TCW+NF+UOCW	Select channels with increased distance from right to left bank including vegetated and wooded islands, with increased ‘openness’ which includes areas without trees located nearby in any direction, and with views unobstructed by dense vegetation or wooded islands.
13	UOCW+UF+UD	Select channels with views unobstructed by dense vegetation or channels with wide unforested widths and amount of flow (cms) per unit of total channel width (m) provides suitable conditions for use.
14	UOCW+UF+DIS	Select channels with views unobstructed by dense vegetation or channels with wide unforested widths and amount of flow (cms) per unit of total channel width (m) provides suitable conditions for use.

All channels throughout the North-central Great Plains within 16 km of a location used by a radio-marked whooping crane were delineated in a GIS. Similar to our previous analysis, we assumed whooping cranes could reasonably evaluate this area based on the field of view they would have at 915 m above ground level. For reasons described above, we used Hawth’s Tools [28] to randomly generate 20 available locations per stopover location within each 32-km river segment. When paired with the use location, each set of points represented a single choice set. Habitat metrics were calculated for each whooping crane use location and 20 corresponding available locations.

A list of 3 candidate models was developed, each containing a different combination of habitat metrics found to influence habitat selection along the central Platte River. Habitat metrics were not included in the same model if their Pearson’s product moment correlation coefficient was high (|r| >0.6). This set of models, with inclusion of a null model containing no habitat metrics, composed the complete set of *a priori* models evaluated (Table 2). Akaike Information Criterion (AIC) statistic was used in the model selection process to determine which *a priori* model was most parsimonious and useful in predicting habitat use [29]. The most parsimonious *a priori* model with a ΔAIC ≤2.0 was used for inference regarding habitat selection [29].

**Table 2:** Description of metrics included in *a priori* model set and tested in the use-availability habitat selection analysis of Great Plains data.

Covariate	Definition of Model Terms
Null	No covariates (habitat selection is random)
UOCW	Unobstructed channel width (m)
NF	Distance to nearest forest (m) truncated at 400 m maximum
UOCW+NF	Unobstructed channel width and distance to nearest forest

### Statistical modeling of habitat selection

Wildlife habitat selection studies with changing availability has received much attention over the last few decades [30–34]. The Platte River ecosystem represents a unique situation in that availability of resources changes on both spatial and temporal scales. The spatial aspect of changing habitat conditions is chiefly due to the variability in channel morphology throughout the 145-km AHR and the temporal component is associated with changes in channel form through time. We chose the discrete-choice method of resource selection function (RSF) estimation to incorporate changing availability at temporal and spatial scales. The discrete-choice model accounts for changing habitat conditions in the study area, while modeling the underlying relationships between selection and predictor variables [34]. Non-linear changes in the RSF due to changing availability were handled with penalized regression splines to approximate the functional response [35].

We used general additive models (GAMs) within a discrete-choice model framework to develop our models. A GAM is a special case of a generalized linear model in which smoothing functions are applied to covariates [36–37]. The model evaluates a weighted relative selection ratio with a multinomial logit form expressed as:

$$w(X_{ij})=exp(s_{1}(X_{1ij})+s_{2}(X_{2ij})+\cdots +s_{p}(X_{pij}))$$ (Eq 1)

where X_1_ to X_p_ were habitat metrics, j indexes the units in the choice set, and i indexes the unit selected, s_1_ to s_p_ were the smooth functions of X_1_ to X_p_, respectively. Relative selection ratios were scaled using the maximum value of the upper confidence interval so that the highest value was one. The smooth terms are penalized regression splines, or smooth functions of the predictor variables describing the relationship between selection and the habitat metrics. Smooth spline functions enabled a wide array of functional forms to be incorporated into the habitat selection model, with the implementation of model selection determining the precise shape of the functional response. The incorporation of penalized regression splines (i.e. smooth terms) into the linear predictor of the model is analogous to the parameterization of a generalized additive model [36]. The smooth term in the habitat model likelihood is represented with a set of basic functions and associated penalties [36–37]. The penalty is larger when the smoothing function is very “wiggly” and requires more degrees of freedom. The degrees of freedom for each smooth term is optimized for each iteration when the likelihood is maximized.

Interpretation of the relationship between metrics in the model and habitat selection was accomplished through response functions. The use-availability likelihood was maximized using R statistical software [38] through RStudio [39], specifically with the gam function of the mgcv package under a Restricted Maximum Likelihood Estimated Cox Proportional Hazards model. The mgcv package determines the smoothness of the spline, and associated degrees of freedom, through iteratively re-weighted least squares fitting of the penalized likelihood [36]. The penalty for the smoothing parameters is determined at each iteration using generalized cross validation. Final model determination among the set of candidate models was obtained using Akaike’s Information Criterion (AIC).

After identifying the best fit model, we estimated the predicted relative selection ratio across the range of observed values of each metric in the model, holding effects of the other variables in the model constant at their means. Interpretation of the relationship between metrics in the model and habitat selection was through response functions and the degrees of freedom for the smooth terms. The estimated degrees of freedom indicate the amount of smoothness, with a value of one equivalent to a straight line. In cases where the estimated degrees of freedom were one, we removed the smoothing component for that covariate and fit a parametric straight line. Due to a small sample size of systematic unique whooping crane group observations, we limited the potential degrees of freedom for regression splines to less than four for all variables. Response functions were scaled to the largest predicted value (maximum equals 1.0) and predictor variables were displayed with 90% confidence intervals from the 10^th^ to 90^th^ percentiles of predictor variables to limit the influence of extreme values on interpretation of results. Point estimates of the predicted relative selection ratios with 90% confidence intervals that overlapped were considered statistically similar. Similar methods were used to develop, identify, and evaluate the top model for the Great Plains data.

### Response functions

Whooping crane habitat use within the AHR has been monitored since 2001. The basic sample unit for this analysis was a crane group (≥1 whooping crane). Per the Program’s systematic monitoring protocol [23], crane groups were identified as being detected systematically during daily monitoring flights. Consequently, this dataset, and associated analyses, was likely more robust as compared to the unequal monitoring effort associated with reports of observations by the public. The first observation of a crane group was identified as being unique with subsequent observations of the same group identified as repeat observations. For example, when crane groups were observed multiple days in a row, only the first observation was considered to be unique (independent).

The model selection process only utilized the first (unique) location for individual crane groups located systematically during implementation of the monitoring protocol [23]. After identifying the best fit model based on the systematic, unique locations, we used all systematically collected locations to estimate the predicted relative selection ratio across the range of observed values of the metrics in the model. This analysis provided a graphical display of the modeled relationship between the predictor variables and the response, holding the effects of the other variables in the model constant at their mean. Graphical displays of response functions were combined with rug plots to show the underlying data in model fitting. Rug plots display a tick mark for each data point in the model, with used points displayed at the top (use equals 1) and the choice set displayed at the bottom of the figure (available equals 0). Response functions were scaled to the largest predicted value (maximum equals 1) and predictor variables were displayed with 90% confidence intervals from the 10^th^ to the 90^th^ percentiles to limit the influence of extreme values on the interpretation of results. We considered overlapping confidence intervals of response function values as statistically similar.

Whooping crane habitat use throughout the north-central Great Plains was monitored from 2010–2016. The basic sample unit for this analysis was a crane group (≥1 whooping crane) consisting of 1 or more radio-marked whooping cranes. Our analyses only utilized the location of each crane group nearest midnight of the first night of each stopover; previous daytime and subsequent daytime and nighttime locations were not included in our analysis. We used these locations to identify the best fit model and to estimate the predicted relative selection ratio across the range of observed values of the metrics in the model. Methods described above were used to display the response functions and data visually.

### Model validation

To validate results of the best model, we randomly partitioned the full dataset of use and corresponding available locations into training (2/3 of the data or 157 choice sets) and test (1/3 of the data or 78 choice sets) datasets. We used training data to develop parameter estimates for best models and a comparison of test dataset available and use locations to understand the reliability of a binary response (use/available) model [40]. Predicted values of available locations within the test dataset were scaled to the number of use locations in the test dataset. These were then binned into twenty percentile categories and compared to the number of test dataset use locations in each bin. Predicted values were summed to calculate the number of expected use locations in each bin, which were then compared to the actual sum of use locations in each bin with a linear regression model to identify the reliability of the model based on the closeness of the slope-relationship of 1. This method was repeated 1,000 times to develop the average slope and 95% confidence intervals of model fit. A “Good” model had an average 95% confidence interval that incorporated 1 and not zero. An “Adequate” model had an average 95% confidence interval that did not incorporated 1 or zero. If the average slope-relationship had a 95% confidence interval spanning zero, the model was deemed “Poor”.

Similar to central Platte River model validation procedures, we randomly partitioned the full dataset of use and corresponding available locations into training (2/3 of the data or 109 choice sets) and test (1/3 of the data or 54 choice sets) datasets. As described above, we evaluated our linear regression model to identify the reliability of the model based on the closeness of the slope-relationship to 1.0.

## Results

### Central platte river

**Fig 4:**
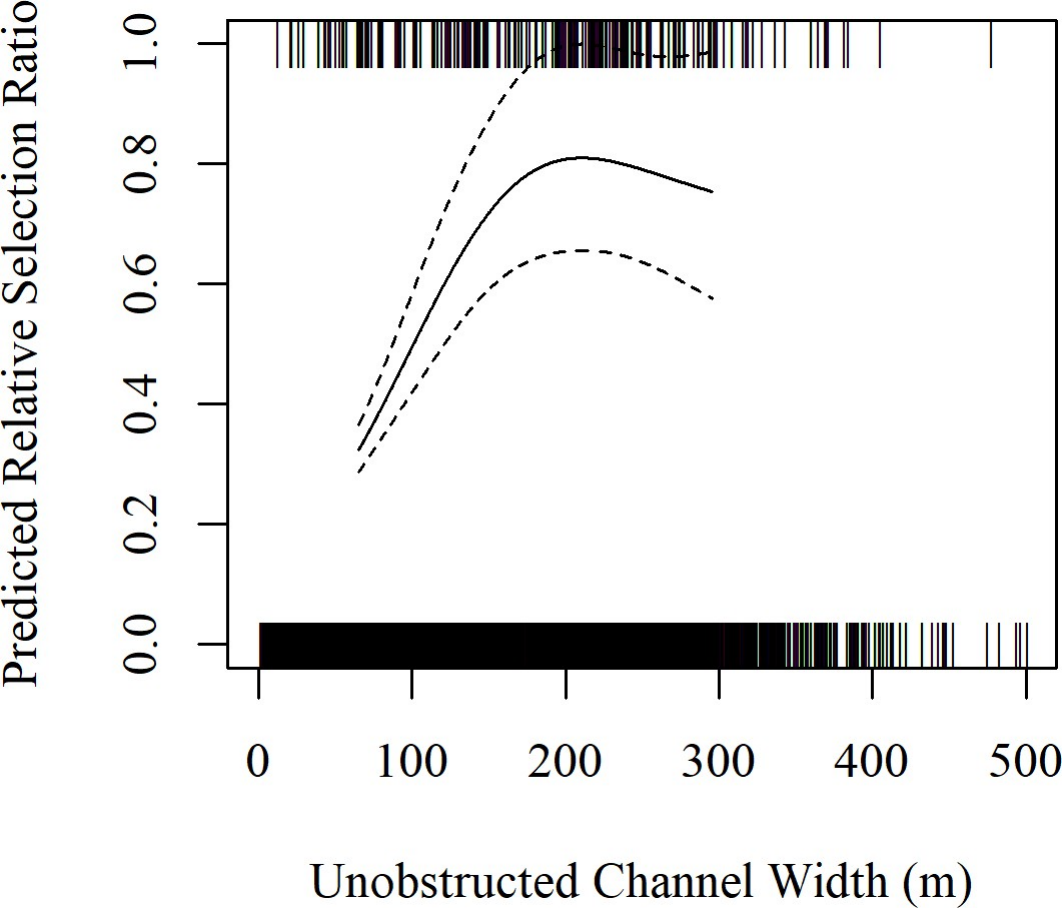
Predicted, relative selection ratio of unobstructed channel width (UOCW) based on all systematically collected whooping crane (n = 235). Tick marks indicate actual data (use points are presented at y = 1 and available points are presented at y = 0). Data is displayed from the 10^th^ to the 90^th^ percentile of use locations with 90% confidence intervals.

**Fig 5:**
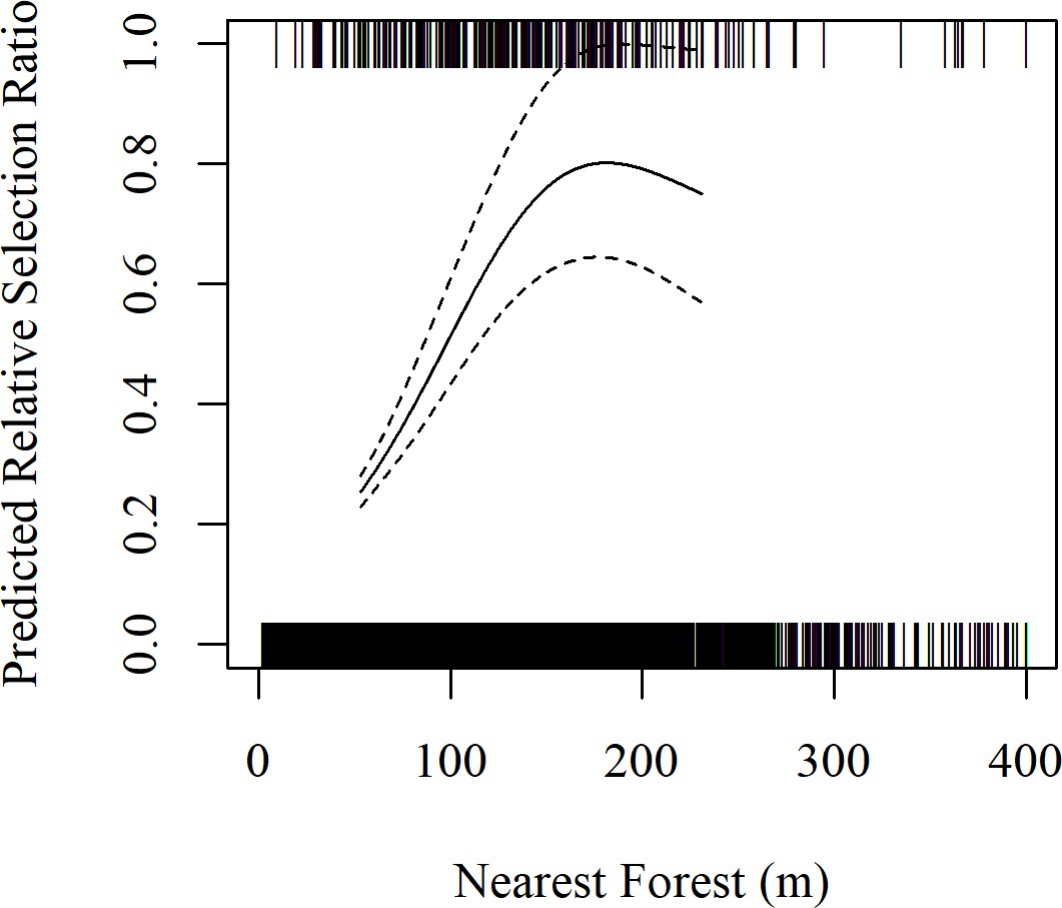
Predicted, relative selection ratio of nearest forest (NF) based on all systematically collected whooping crane roost locations (n = 235). Tick marks indicate actual data (use points are presented at y = 1 and available points are presented at y = 0). Data is displayed from the 10^th^ to the 90^th^ percentile of use locations with 90% confidence intervals.

Data obtained from systematic aerial surveys of our study area over 32 migration seasons from spring 2001– spring 2017 (no surveys were conducted during fall 2003) provided 85 systematic, unique use locations and 235 systematically collected use locations. The 235, systematic whooping crane group observations included the 85 unique locations as well as 150 subsequent observations of the 85 unique whooping crane groups observed during aerial surveys. In-channel riverine habitat selection models were developed for the 85, systematic unique whooping crane group observations and the associated 1,700 available points. Statistical modeling of habitat use indicated UOCW and NF were the most important predictors of selection of in-channel riverine habitat (Table 3). Based on detection trials, we also found parameters in our final model did not influence detection rates and thus our model was considered robust to any potential detection biases. We used this model to analyze the 235 systematically collected whooping crane group observations identified during aerial surveys as well as the associated 4,700 available points. Model results indicate UOCW and NF relationships were similar to results of models derived from the systematic unique dataset, but the confidence in estimates were tighter because the sample size was increased from 85 to 235 use locations. The relative selection ratio was maximized at an UOCW of 210 m, but relative selection ratios were statistically similar for UOCW’s larger than 110 m (Fig 4). Similarly, the relative selection ratio was maximized at 181 m from the nearest forest, but relative selection ratios were statistically similar for distances larger than 104 m (Fig 5). The estimated degrees of freedom for the smoothed terms were 3.0 for UOCW and 3.1 for NF. A good model fit was indicated as the slope and 95% confidence interval of the model validation relationship averaged 0.894 (95% CI = 0.607–1.182).

**Table 3:** In-channel riverine habitat use model selection for whooping crane group stopover sites on the central Platte River. AIC value of null model was 1,406.62.

Model	Metrics	df	AIC	ΔAIC	weight
13	UOCW+NF+UD	93.28	1,359.05	0.00	0.44
8	UOCW+NF	90.59	1,360.40	1.35	0.23
14	UOCW+NF+DIS	91.54	1,360.57	1.51	0.21
12	TCW+UOCW+NF	91.59	1,361.83	2.78	0.11
4	NF	87.34	1,366.10	7.05	0.01

### North-central great plains

**Fig 6:**
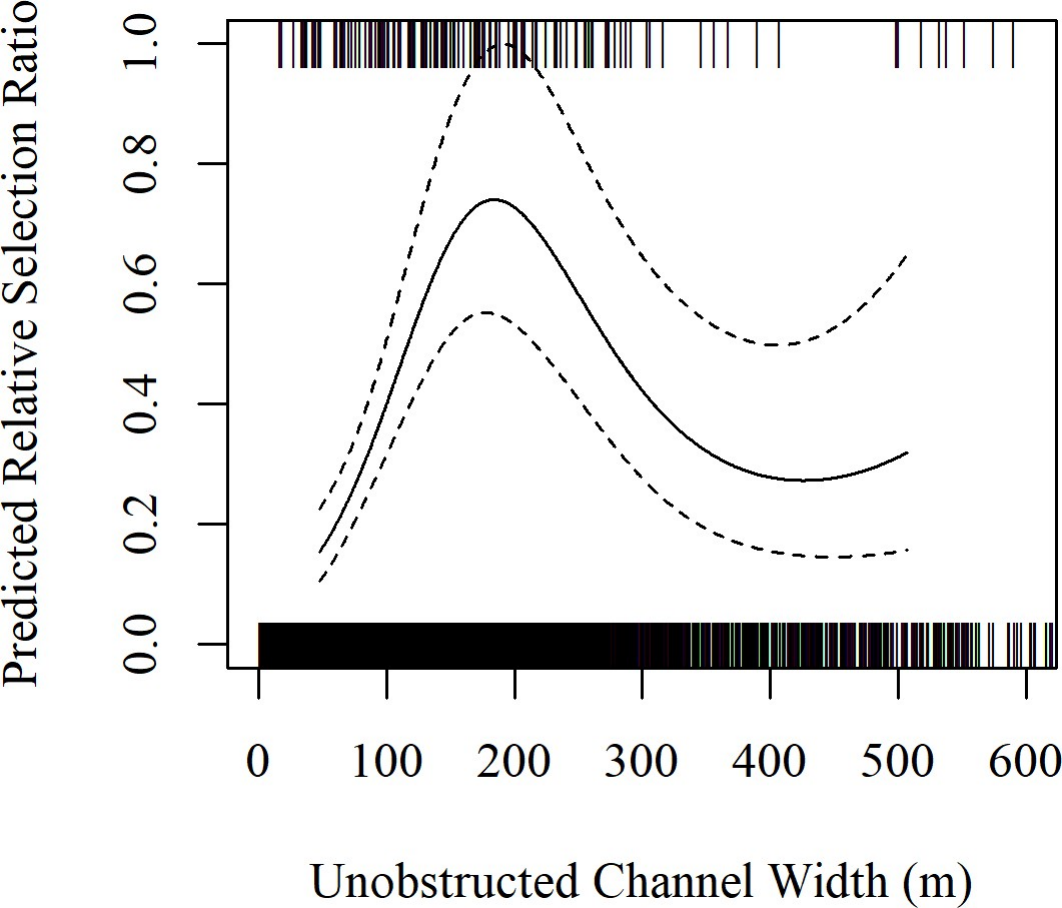
Predicted relative selection ratio (solid line), with 90% confidence intervals (dashed lines), between the 10^th^ and 90^th^ percentiles of unobstructed channel widths (UOCW). Tick marks display response data (use locations are plotted at y = 1; available locations are plotted at y = 0). One use and 56 available locations that ranged in width from 1,054 m to 2,189 m are not included in the plot.

**Fig 7:**
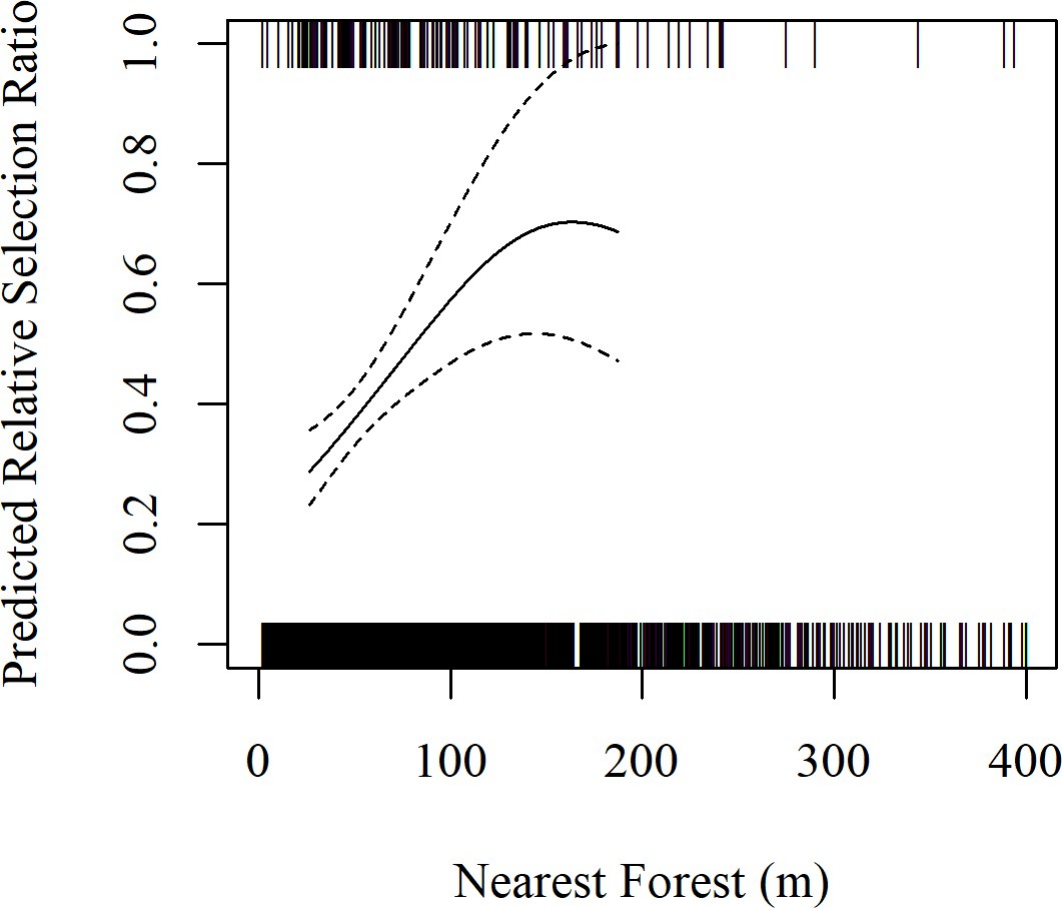
Predicted relative selection ratios (solid line) with 90% confidence intervals (dashed lines) between the 10^th^ and 90^th^ percentiles of distance to nearest forest. Tick marks indicate response data (use locations are at y = 1, available locations are at y = 0).

Telemetry data obtained from a sample of 39 birds of all ages provided 147 independent stopover locations over 12 migration seasons, 2010–2016. Statistical modeling of habitat selection indicated UOCW and NF were also important predictors of whooping crane riverine habitat selection throughout the North-central Great Plains (Table 4). Predicted relative selection ratios increased with UOCW and were maximized at 184 m; however, relative selection ratios were statistically similar for UOCW’s ranging from 105 m to 465 m (Fig 6). Predicted relative selection ratios also increased with NF and were maximized at 164 m and relative selection ratios were statistically similar for NF ranging from 67 m to >400 m (Fig 7). The estimated degrees of freedom for the smoothed terms were 6.7 for UOCW and 2.7 for NF. An adequate model fit was indicated as the slope and 95% confidence interval of the model validation relationship averaged 0.469 (95% CI = 0.039–0.900).

**Table 4:** *A priori* models used in habitat selection analysis ranked by AIC statistic. See Table 2 for a description of the metrics use in our Great Plains data analysis.

Rank	Metrics	df	AIC	ΔAIC	weight
1	UOCW + NF	156.62	2,614.87	0.00	0.95
2	UOCW	153.38	2,620.86	6.00	0.05
3	NF	149.73	2,638.10	23.23	0.00
4	NULL	146.00	2,654.28	39.41	0.00

## Discussion

We evaluated riverine roost sites, which account for approximately 20% of sites used by migrating whooping cranes to identify habitat characteristics important to selection of used sites that could be managed by conservation organizations. We confirmed certain key resource features related to openness of the roost site but were unable to establish links between roost site selection and flow metrics as have been observed previously. Whooping cranes, sandhill cranes, and other species of wading birds have been found to select for open roost sites [41–46]. Perceived security from predators has been speculated as motivation to select open sites, allowing the birds to detect threats and react appropriately [42]. Metrics related to river flow and water levels have been used to index amount of shallow water in a defined area usable by cranes. Therefore, site choice for whooping cranes may more limited by perception of safety from predators than amount of physical space available in river reaches.

Evaluations of habitat characteristics at whooping crane roost date to the early 1980s [4,8,10,12,16,18–22,42–44]. Several characteristics commonly associated with whooping crane riverine roost sites include shallow, wide, unvegetated channels and open visibility with the absence of tall trees or dense shrubs near the roost [4,10–12,14,18,20–22,44–46]. Our results support these characterizations as UOCW and NF were found to be important predictors of whooping crane group roost site selection. Riparian forests are common features associated with rivers in the Great Plains. Austin and Richert [16] reported >70% of whooping crane roost sites were adjacent to woodland habitat, highlighting the common nature of these features. Whooping cranes roosting on the Platte River have been noted to select sites relatively free of woody vegetation allowing for horizontal and overhead visibility [21]. Our results suggest riparian forests <160 m from a potential roost area negatively influenced selection of river reaches. As with unobstructed channel width, we found this effect moderated with increasing distances, such that riparian forest had little additional influence on relative probability of use when >160 m away. However, it has also been suggested that physical and vegetative obstructions may enhance perceived security from disturbance features such as roads, as long as visual obstructions were not too close to the cranes [18,47]. Our results generally support both the notion that whooping cranes perceived riparian forests as a negative factor to a threshold distance and were of little consequence at greater distances.

Unobstructed bank-to-bank width has been a common metric used to characterize visibility at river roost sites. Universally, studies and reports suggest that narrow channels provide poor and wide channels provide preferred roost habitat for whooping cranes [11,20,45,46]. To date, however, roost characteristics and criteria have generally been developed based on a limited amount of quantitative information and most criteria have been derived from qualitative assessments and circumstantial roost locations that may not be representative of typical stopover sites [18]. Unobstructed channel widths of ≤75 [45] and ≤152 m [46] have been suggested to be unsuitable for roost sites; however, these estimates were based on opinion rather than an analysis of data. We used two different datasets to provide independent and robust evaluations of whooping crane use and selection generally supports some of these past conclusions. We found that relative probability of use increased dramatically as unobstructed channel width increased until approximately 150 m in both analyses. Yet, 50% of roost sites across a large segment of the migration corridor in the United States were in channels with unobstructed widths that were <166 m and average width was 230 m. Thus, our results support the notion that whooping cranes have decreased probability of using river reaches that are narrow (i.e., ≤150m), yet they regularly used these narrower sites, likely because wider and more preferred sites may not have been available to them.

Estimates and recommendations for unobstructed channel values that constitute optimal river roost sites for whooping cranes has varied between 351–400 m [12,44,45,46,48]. Similarly, Lingle et al. [49] suggested whooping cranes choose the widest sites available to them. However, Johnson [10] described optimal riverine roost habitat as being any channel with an unobstructed width ≥155 m, which is similar to our findings. Our results indicated that channels of widths approaching 200 m maximized relative probability of use and that values above may provide little marginal gains. We interpret our results of distance to riparian forest in a similar manner, where we find no evidence of additional increases in probability of roost site use at distances >160 m. River reaches that exceed these estimated thresholds are highly preferred by whooping cranes, yet whooping cranes seemingly do not perceive additional benefits to locations that appreciably exceed these parameters. Although, benefits perceived by whooping cranes may be maximized at these threshold channel widths and forest values, fitness benefits as determined from increased probability of survival were not estimated because we did not detect deaths of cranes at any river roost site.

Moreover, roost-site selection results provided evidence that relative probability of use does not increase at channel widths >200 m, suggesting that benefits perceived by whooping cranes may be maximized at these unobstructed channel widths. Austin and Richert [22] found river widths at stopover roost locations distributed throughout the migration corridor ranged from 76 m to 457 m and averaged 233 m whereas Faanes et al. [18] found unobstructed channel widths observed at roost sites on the Platte River averaged 217 m. Though river widths reported by Austin and Richert [22] are slightly wider than unobstructed channel widths we observed, discrepancies in these measures could simply be an artifact of how each metric was defined (i.e., river width may not be comparable to unobstructed channel width).

Past research has indicated whooping cranes tend to select roost habitat with increased wetted width and area of suitable depth [8,45]; we however, did not find a relationship between roost site selection and the flow metrics we evaluated. Unit discharge is related to flow, wetted width, and area of suitable depth in that an increase in unit discharge (increase in flow or decrease in channel width) would generally equate to an increase in wetted width and a decrease in area of suitable depth. A strong relationship between unit discharge or discharge divided by wetted channel width and whooping crane use was found by Biology Workgroup [8] and Farmer et al. [44]. Our analysis, however, did not identify a strong relationship between flow-related metrics and whooping crane use location. The lack of a strong relationship between flow metrics and whooping crane use location can be interpreted 2 ways: 1) flow is not important in whooping crane selection of a roost location, or 2) sufficient areas of suitable depth and wetted area were equally available and adequate at use and available locations on use days. However, it should be noted that our analysis only addressed flow within the context of roost location choice, not the decision to stop or not stop and use riverine habitat based on flow conditions. Such an analysis would need to include absence data which would require us to know flow conditions when whooping crane groups chose not to roost within a river site; however, those data are not available. Given water is almost always associated with whooping crane roost locations, it is likely that sufficient areas of suitable depth and wetted area were available at use and available locations, reducing the importance of flow-habitat metrics in roost site selection. A crane group comprised of four to six individuals will roost in an area that is generally less than 15 m by 15 m (David Baasch, personal observations). Under most flow and channel configuration combinations, there is much more shallow water (<25 cm) suitable for roosting habitat than is required to accommodate these sizes of crane groups.

We conducted analyses specifically to support management of the Platte River and, more generally, other river systems in the Great Plains. We focused on river roosts solely because of this primary focus and rivers have certain unique characteristics not directly relevant to other surface water types such as channel width and flowing water. Although, palustrine and lacustrine wetlands represent the most common roost sites used by migrating whooping cranes [2]. Although results may not be directly applicable to management of these important resources, they may provide select insights to all potential roost sites during migration or other times of the year. We found that characteristics related to perceived security of sites may have motivated cranes to select certain sites over others compared to amount of shallow water available at a site as indexed by river flow metrics. Hence, promoting creation or management of open sites may be of greater value than those with large amounts of shallow water. In addition, we found that providing ever increasing amounts of open space may have diminishing returns in attractiveness of the site for whooping cranes. Although the relationship between openness and habitat selection likely varies with types of surface water features, acknowledgment of potential thresholds may have value in understanding roost site selection for whooping cranes in other situations.

## Conclusions

Several studies have characterized habitat use by whooping cranes using the U.S. Fish and Wildlife Service’s opportunistic sightings database [7,16–17,19]. These characterizations, however, may have been influenced by sampling bias, detection bias, and location error inherent in these data [7]. Thus, we used data collected systematically along the central Platte River during 2001–2017 to evaluate riverine habitat selection within the AHR. The goal of our analysis was to develop habitat models to inform and direct management activities the Program is able to implement. We were unable to establish a relationship between whooping crane use and flow metrics or total channel width, but rather found unobstructed channel width and distance to the nearest forest were the top predictors of whooping crane use. We found the positive association between unobstructed channel width and distance to nearest forest waned at moderate metric values (210 m and 180 m, respectively). Next, we used telemetry data obtained from a sample of 39 birds of all ages over the course of seven years to provide 147 independent stopover locations which allowed access to a substantial set of robust data to evaluate whooping crane use of riverine habitat throughout the migration corridor. Similar to our central Platte River evaluation, we found a positive response for increasing unobstructed channel widths and distances to nearest forest (i.e., narrow or small values resulted in lowest selection ratios). However, similar to habitat selection along the central Platte River, selection ratios were again generally maximized at moderate metric values. Thus, it appears the influence of each of these metrics on selection of river reaches abates at some modest values in comparison with available sites, rather than whooping cranes selecting the widest stretch of river available devoid of trees as has been suggested in previous analyses and efforts. Therefore, our results suggest maintaining unobstructed channel widths of 200 m and unforested corridor widths of 330 m throughout the migration corridor would result in highly favorable whooping crane riverine roosting habitat. From a management perspective, our findings indicate resource managers, such as the Program, may be able to influence whooping crane use of riverine habitat through increasing unobstructed channel widths that are <200 m and mechanically removing trees within areas where the unforested corridor width is <330 m. With selection ratios seemingly maximized at these unobstructed channel and unforested widths, managing for sites with wider characteristics would likely increase costs without realizing additional perceived or appreciable benefits to whooping cranes.
